# Enhancement of the Colorectal Chemopreventive and Immunization Potential of Northern Thai Purple Rice Anthocyanin Using the Biotransformation by β-Glucosidase-Producing *Lactobacillus*

**DOI:** 10.3390/antiox11020305

**Published:** 2022-02-02

**Authors:** Sasithorn Sirilun, Chaiyavat Chaiyasut, Thanawat Pattananandecha, Sutasinee Apichai, Jakkapan Sirithunyalug, Busaban Sirithunyalug, Chalermpong Saenjum

**Affiliations:** 1 Department of Pharmaceutical Sciences, Faculty of Pharmacy, Chiang Mai University, Chiang Mai 50200, Thailand; ssirilun@gmail.com (S.S.); chaiyavat@gmail.com (C.C.); thanawat.pdecha@gmail.com (T.P.); sutasinee.apichai@gmail.com (S.A.); jakkapan.s@cmu.ac.th (J.S.); 2 Innovation Center for Holistic Health, Nutraceuticals and Cosmeceuticals, Faculty of Pharmacy, Chiang Mai University, Chiang Mai 50200, Thailand; 3 Center of Excellence for Innovation in Analytical Science and Technology for Biodiversity-Based Economic and Society (I-ANALY-S-T_B.BES-CMU), Chiang Mai University, Chiang Mai 50200, Thailand; 4 Multidisciplinary Approaches to Lanna Fermented Foods and Biological Resources Research Unit, Sciences and Technology Research Institute (STRI), Chiang Mai University, Chiang Mai 50200, Thailand

**Keywords:** Thai purple rice anthocyanin, biotransformation, β-glucosidase-producing *Lactobacillus* (BGPL), colorectal chemoprevention, immunomodulatory, natural active pharmaceutical ingredient (NAPI)

## Abstract

This study aimed to study the biotransformation of indigenous northern Thai purple rice using β-glucosidase-producing *Lactobacillus* (BGPL) to increase the content of bioactive anthocyanin for colorectal chemoprevention and immunization. BGPL, namely, *Lactobacillus* FR 332, was first isolated from Thai fermented foods. Indigenous northern Thai purple rice, namely, Khao’ Gam Leum-Phua (KGLP), was selected to study bioactive anthocyanin using biotransformation by *L. plantarum* FR332 according to the highest amounts of cyanidin-3-glucoside. The determination of anthocyanin quantities revealed that the highest cyanidin was detected after 12 h of biotransformation, corresponding to the highest β-glucosidase activity of *L. plantarum* FR332 and a decrease in cyanidin-3-glucoside. The anthocyanin extract, after 12 h of biotransformation, exhibited the most potent in vitro antioxidative activity. Additionally, it showed potent anti-inflammatory activity by inhibiting cyclooxygenase-2 (COX-2), nitric oxide, and inducible nitric oxide synthase (iNOS) production in interferon-γ-stimulated colon adenocarcinoma (HT-29) cells without exerting cytotoxicity. Moreover, it also showed a potent inhibitory effect on proinflammatory cytokine interleukin-6 (IL-6) secretion and an induction effect on anti-inflammatory cytokine IL-10 secretion. These documents highlight the potential to be used of the anthocyanin extract after 12 h of biotransformation by *L. plantarum* FR332 as a natural active pharmaceutical ingredient (NAPI) for colorectal chemoprevention and immunization.

## 1. Introduction

Carcinogenesis is influenced by inherited and acquired susceptibility factors, initiation factors including external and endogenous carcinogens, and promotion and progression factors. Chemoprevention through the use of drugs, natural or synthetic active pharmaceutical ingredients (APIs), allows the suppression, retardation, inhibition, or intervention of carcinogenesis, which may be conducted at a variety of timepoints of carcinogenesis to prevent induction and inhibit or delay the progression of cancers [1,2]. It has been reported that numerous natural active pharmaceutical ingredients (NAPIs) can protect against cell proliferation in the destruction of certain reactive oxygen and reactive nitrogen species (ROS and RNS), which initiate carcinogenesis through oxidative and nitrosative damage to DNA. Various NAPIs present in the human diet, such as curcumin, resveratrol, flavonoids, polyphenols, and anthocyanins, have been identified as potential chemopreventive and/or chemotherapeutic agents [3,4]. The prevalence of colorectal cancer is increasing, with more than one million people worldwide suffering from this disease. It has been reported that using cyclooxygenase-2 (COX-2) inhibitors, namely, aspirin and celecoxib, can reduce the risk of colorectal cancer in high-risk groups [5,6]. However, aspirin and celecoxib are limited in patients with gastric ulcers or a high risk of bleeding, and they are contraindicated in patients with chronic kidney failure. Additionally, the selective COX-2 inhibitor celecoxib is precautioned in patients with cardiovascular disease.

Anthocyanins are natural, water-soluble plant pigments which occur in red, purple, and blue colors, and their color changes depending on pH [7]. Anthocyanins are the most significant subgroup of flavonoids commonly found in flowers, vegetables, and fruits. Anthocyanins are usually found in glycoside and acylated forms [7]. They are composed of anthocyanidin, known as aglycone, which is attached to mono-, di-, or triglycosides via an α- or β-linkage at C-3 of the aglycone [8]. It has been shown that the biological activities of anthocyanin promote human health, including anticancer, antidiabetic, anti-inflammatory, antimicrobial, anti-obesity, and antioxidant activities [7,9]. However, the effectiveness of anthocyanin in the protection of human health is influenced by its bioavailability. Evidence suggests that the molecular size and the acylated group attached may influence absorption [8,10,11,12]. Glycosylation also affects the biological activity of anthocyanin, which makes the molecule more soluble in water [13]. The rate and the extent of absorption are also affected by the glycone, sugar moiety, and acylated group [14]. 

Intestinal bacteria, especially lactic acid bacteria (LAB), including *Lactobacillus* spp. and *Bifidobacterium* spp., have been shown to exert beneficial effects on the human gastrointestinal tract by balancing the intestinal microbiota and producing various beneficial metabolites to support the biotransformation process as a plant starter culture, including the β-glucosidase enzyme which is capable of hydrolyzing various flavonoid glycosides [15,16,17]. The β-glucosidase enzyme hydrolyzes plant glycosides into a non-sugar residue (aglycone), which is a bioactive aromatic compound [18,19]. Leal-Sanchez [20] reported that β-glucosidase-producing *Lactobacillus* (BGLP) strains have the potential to hydrolyze some plant-derived compounds, such as debittering olives by fermentation and transforming soy isoflavones into aglycones, which are connected to cancer prevention and the relief of menopausal symptoms [18], inflammation, and cardiovascular disease [21]. Thus, the administration of BGLP in fermented food may lead to the incorporation of functional health benefits, as well as the bioconversion or biotransformation of glycosides to bioactive aglycones.

Thai purple rice is commonly cultivated and consumed by the Thai population, especially in the mountainous area in northern Thailand. It is also popular for maintaining one’s health and preventing various diseases. Purple rice is rich in health-beneficial phytochemicals such as γ-oryzanol, tocotrienols, tocopherols, phenolic compounds, and anthocyanins, especially cyanidin-3-glucoside and peonidin-3-glucoside [22,23,24]. The present study was carried out to study the biotransformation of indigenous northern Thai purple rice containing cyanidin-3-glucoside using BGLP isolated from Thai fermented foods. A kinetic study of bioactive anthocyanins and their biological activity for colorectal chemoprevention and immunization was also conducted by determining their inhibition effect on nitric oxide (NO), inducible nitric oxide synthase (iNOS), and COX-2 in interferon-γ (IFN-γ)-stimulated colon adenocarcinoma (HT-29) cells. Moreover, the effects on proinflammatory cytokine interleukin-6 (IL-6) and anti-inflammatory cytokine IL-10 were investigated. The current results may establish the health benefits of biotransformed Thai purple rice and support its use as a NAPI in nutraceutical or pharmaceutical products.

## 2. Materials and Methods

### 2.1. Materials

Cyanidin-3-glucoside, delphinidin-3-glucoside, peonidin-3-glucoside, pelargonidin-3-glucoside, malvidin-3-glucoside, cyanidin chloride, delphinidin chloride, peonidin chloride, pelargonidin chloride, and malvidin chloride were purchased from Extrasynthese Co., Ltd. (Genay, France). Curcumin, quercetin, and l-ascorbic acid were purchased from Tokyo Chemical Industries Co., Ltd. (Tokyo, Japan). All solvents and chemicals used were either analytical or HPLC grade and were obtained from Sigma Chemical Co., Ltd. (St. Louis, MO, USA) and Merck (Darmstadt, Germany). All chemicals, media, and solvents used in the cell-based study were obtained from Invitrogen^TM^ (Carlsbad, CA, USA) and Roche (Mannheim, Germany). Colorectal adenocarcinoma cells (HT-29) were purchased from the American Cell Culture Collection (Bethesda, MD, USA). The COX-2, iNOS, IL-6, and IL-10 immunoassays were purchased from R&D System (Minneapolis, MN, USA).

### 2.2. Selection of Indigenous Northern Thai Purple Rice

Five native indigenous northern Thai purple rice cultivars were harvested from a rice research center in Northern Thailand. The purple rice cultivars, namely, Khao’ Gam Boung (KGB), Khao’ Gam Thor (KGT), and Khao’ Gam Leum-Phua (KGLP), were collected from the Mae Hong Son rice research center, Mae Hong Son, Thailand. Khao’ Gam Pha Yao (KGPY) and Khao’ Gam Chiang Rai (KGCR) were collected from the Chiang Rai Rice Research Center, Chiang Rai, Thailand. The Thai purple rice was dried in a hot air oven at 60 °C for 24 h. Then, the purple rice samples were milled using a rice milling machine (Natrawee Technology Co., Ltd., Chachoengsao, Thailand, Model: NW 1000 TURBO). The anthocyanin extract of the purple rice samples was prepared using alcoholic water, pH 2.0 (80% ethanol), and the remaining solvent was evaporated under reduced pressure and dried under vacuum. All samples were analyzed for anthocyanins by reverse-phase high-performance liquid chromatography (RP-HPLC) with slight modifications from Pengkumsri et al. [25], using an Agilent 1200 equipped with a multiwavelength detector. The mobile phase consisted of 3% phosphoric acid in acetonitrile and deionized water at the flow rate of 1.0 mL/min. A Symmetry RP18 Column (4.6 mm × 250 mm), 5 µm particle diameter, Waters Co., Ltd. (Milford, USA)) was used to separate each form of the anthocyanins. The detection wavelength was set to 520 nm. The linear gradient elution was operated from 0 to 40 min, with acetonitrile between 10% and 20%. All samples were tested in triplicate (*n* = 3). The purple rice containing the highest cyanidin-3-glucoside was selected to study the kinetic biotransformation using β-glucosidase-producing *Lactobacillus* (BGPL).

### 2.3. Selection of β-Glucosidase-Producing Lactobacillus Strain

The *Lactobacillus* strains were isolated from Thai fermented foods. All isolates were cultured and purified onto modified de Man Rogosa Sharpe (MRS) agar (10 g of purple rice powder, 10 g of peptone, 5 g of yeast extract, 1 mL of Tween-80, 2 g of ammonium citrate dibasic, 5 g of sodium acetate, 0.1 g of MgSO_4_·7H_2_O, 0.05 g of MnSO_4_·H_2_O, 0.5 g of bromocresol purple, and 15 g of bacto agar, in a total volume of 1 L), before being aerobically maintained at 37 °C for 24–48 h. The isolated strains were stored in 20% (*v*/*v*) glycerol in MRS at −70 °C. Then, 4% (*v*/*v*) of each strain was inoculated into 10 mL of modified MRS broth for 10 timepoints (0, 6, 12, 18, 24, 36, 48, 72, 120, and 168 h) at 37 °C. The supernatants of the culture strains were collected by centrifugation at 5000× *g* for 10 min at 4 °C to determine enzyme activities. The β-glucosidase activity was determined by measuring the rate of the hydrolysis of *p*-nitrophenyl-β-d-glucopyranoside (pNPG) according to the method of Otieno et al. [26]. Briefly, a concentration of 5 mM pNPG dissolved in 0.5 M sodium phosphate buffer (pH 7.0) was added to cell-free supernatant, and then the mixture was incubated for 30 min at 37 °C. The reactions were stopped by adding 2.5 M cold sodium carbonate (4 °C), before immediate measurement at 405 nm using a multimode spectrophotometer. One unit of the enzyme activity was calculated as the quantity of β-glucosidase that released 1 µmol of *p*-nitrophenol from pNPG per minute under the assay conditions.

### 2.4. Stability of β-Glucosidase Enzyme Activity at Various pH and Temperature

The effects of different initial pH values and incubation temperatures on the enzyme activities were determined. The assay was a modification of the method of Michlmayr et al. [27]. The initial pH of the sodium phosphate buffer used in the reaction mixture was adjusted to 2.0, 2.5, 3.0, 3.5, 4.0, 4.5, 5.0, 5.5, 6.0, 6.5, and 7.0. The incubation temperatures of reactions were varied to 4, 15, 25, 30, 37, 42, 50, and 55 °C at the optimal initial pH.

### 2.5. Purple Rice Anthocyanin Biotransformation Study

A total of 598 *Lactobacillus* isolates were obtained from 247 samples of selected Thai fermented foods. The highest activity of β-glucosidase was detected in the culture supernatant of the *Lactobacillus* FR 332 strain. The *Lactobacillus* FR 332 strain was inoculated in medium overnight and its genomic DNA was identified via comparison with 16S rDNA sequence analysis, which identified the specie as *L. plantarum* [28]. Briefly, the 16S rDNA primers 5′–GCCGCCTAAGGTGGGACAGAT–3′ (forward primer) and 5′–TTACCTAACGGTAAATGCGA–3′ (reverse primer) [29] were amplified using a PCR reaction, and then submitted to the KU-Vector Custom DNA Synthesis Service, Kasetsart University, Bangkok, Thailand, for sequencing. The BLASTN program (http://blast.ncbi.nlm.nih.gov/blast.cgi (accessed on 10 December 2012)) was used to conduct a homology search of the 16S rDNA in the National Center for Biotechnology Information (NCBI) GenBank. The purple rice samples were collected at 0, 6, 12, 18, 24, 36, and 48 h, as well as at 3, 5, 7, 10, 14, 17, and 21 days, of the biotransformation process by selected BGPL. All samples were dried at 50 °C for 24 h. Then, 100 g of dried biotransformation purple rice samples was extracted using hydroalcoholic pH 2.0, evaporated under reduced pressure, and dried under vacuum. The obtained extracts were analyzed for each form of anthocyanin by reversed-phase HPLC as previously described.

### 2.6. Effect of Biotransformed Anthocyanin on In Vitro Antioxidant Activity

#### 2.6.1. 2,2′-Azino-bis-3-ethylbenzothiazoline-6-sulfonic Acid (ABTS) Radical-Scavenging Assay

The ABTS free-radical scavenging assay was also performed, using the method of Promnoi et al. [30] with slight modifications. The ABTS solution was prepared in potassium persulfate and stored in the dark for 12–14 h. Then, the prepared solution was diluted with deionized water to get an absorbance at 734 nm of approximately 0.70–0.75. The experimental solution consisted of 10 μL of various concentrations of tested samples and 990 μL of working ABTS solution. The reaction mixture was incubated for 5 min in the dark. The absorbance was measured at 734 nm. l-ascorbic acid, cyanidin-3-glucoside, and cyanidin chloride were used as the positive control. The results were calculated and expressed as 50% ABTS decolorization (IC_50_).

#### 2.6.2. Superoxide Anion Radical-Scavenging Assay

The scavenging effects on the superoxide anion of the tested samples were determined according to the method of Saenjum et al. [31]. Initially, superoxide anion radicals were generated in a β-nicotinamide adenine dinucleotide (NADH)–phenazine methosulfate (PMS) system through the oxidation of NADH and then analyzed by the reduction of nitroblue tetrazolium (NBT). The reaction mixture was prepared in PBS buffer (pH 7.4), containing 78 μM NADH, 25 μM NBT, 45 μM EDTA, and different concentrations of the positive controls (L-ascorbic acid, cyanidin-3-glucoside, and cyanidin chloride) or the tested samples. Then, PMS was added to initiate the reaction, and, after 5 min of incubation in the dark, the absorbance was measured at 560 nm. All samples were tested in triplicate (*n* = 3). The results were calculated and expressed as a 50% inhibition concentration (IC_50_).

#### 2.6.3. Nitric Oxide Radical-Scavenging Assay

In vitro NO scavenging activity was assayed using the Griess reaction described in Pengkamsri et al. [32] with slight modifications. Briefly, 6.25 M of sodium nitroprusside was prepared in PBS buffer and different concentrations of the positive controls (curcumin, cyanidin-3-glucoside, and cyanidin chloride) or the tested samples. The reaction mixtures were incubated at 37 °C for 150 min. After the incubation period, the reaction mixture was transferred to a 96-well plate. The Griess reagent, which consisted of naphthylethylene diamine and sulfanilamide, was added and incubated at room temperature for 5 min. The absorbance was measured at 540 nm. All samples were tested in triplicate (*n* = 3). The results were calculated and expressed as IC_50_.

### 2.7. Effect of Biotransformed Anthocyanin on Colorectal Chemopreventive and Immunization Potential in HT-29 Cells

The effects on nitric oxide, iNOS, COX-2, IL-6, and IL-10 production were investigated using the improved methods of Hong et al. [33], Sirithunyalug et al. [34], and Phromnoi et al. [35]. The effects of the selected biotransformed anthocyanin on nitric oxide, iNOS, COX-2, IL-6, and IL-10 production were assayed using a human total iNOS, COX-2, IL-6, and IL-10 immunoassay. The HT-29 cells (5 × 10^5^ cells/well) were preincubated in 24-well plates for 24 h. Then, the medium was replaced with fresh medium containing tested samples in the concentration of 10–100 µg/mL, the tested concentrations were selected from HT-29 cells viability cultured without and with interferon-γ (IFN-γ) as shown in Figures S1 and S2, respectively. After 12 h of incubation, IFN-γ was added and left to incubate for a further 72 h. The culture medium supernatants were collected for analysis of nitric oxide, IL-6, and IL-10 levels. Moreover, cells were lysed to yield cell lysates using CelLytic^TM^ M Cell Lysis Buffer (Sigma, C2978) to perform the assays for iNOS and COX-2. The nitrite level in the culture medium was measured using Griess reagent as an indicator of nitric oxide production. The absorbance was measured at 540 nm against a standard curve of potassium nitrite [36]. IL-6 and IL-10 were also measured using commercially available human IL-6 and IL-10 ELISA kits following the manufacturer’s protocol. Additionally, commercially available human iNOS and COX-2 ELISA kits were used to measure the production of iNOS and COX-2 in the cell lysates. Cyanidin chloride, cyanidin-3-glucoside, and curcumin (2.5, 5, 10, and 25 µg/mL) were used as the positive controls. The selected purple rice extract without the biotransformation process was investigated for comparison to its biotransformed counterpart. The protein produced by HT-29 cells was analyzed using Bradford reagent (Sigma Chemical Co., Ltd., St. Louis, MO, USA). Concurrently, the viability of HT-29 cells with and without stimulation with IFN-γ was assayed according to the improved methods of Sirithunyalug et al. [34] in the absence or presence of tested samples for 72 h using the cell proliferation reagent WST-1 (Roche, Basel, Switzerland).

## 3. Results

### 3.1. Selection of Thai Purple Rice

The amounts of analyzed anthocyanins in the purple rice extracts are shown in Table 1. Cyanidin-3-glucoside and peonidin-3-glucoside were the predominant anthocyanins found in all the purple rice extracts; cyanidin was found in KGLP, KGB, and KGT, while peonidin was not detected. The results showed that KGLP had the significantly highest (*p* < 0.05) content of cyanidin-3-glucoside and peonidin-3-glucoside (192.19 ± 3.10 and 84.54 ± 2.78 mg/100 g dried weight), followed by KGB (108.49 ± 2.78 and 57.83 ± 2.19 mg/100 g dried weight). Therefore, KGLP was selected to study the biotransformation by BGPL according to the highest content of cyanidin-3-glucoside.

### 3.2. Kinetics of Anthocyanin Content during Biotransformation Process

**Figure 1:**
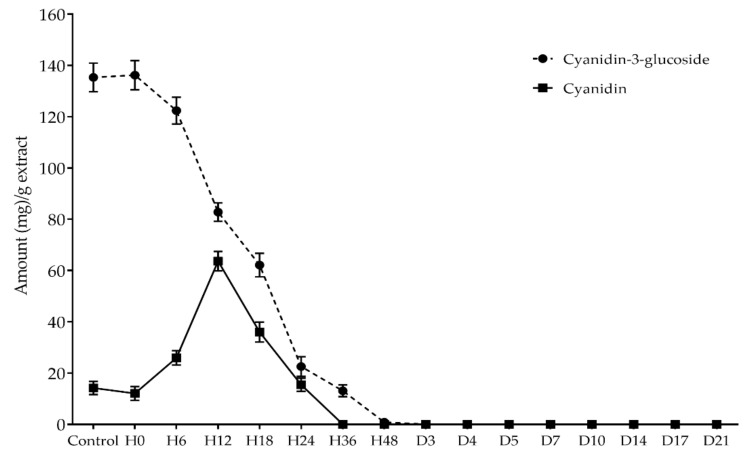
Kinetic study of cyanidin-3-glucoside and cyanidin during biotransformation process.

**Figure 2:**
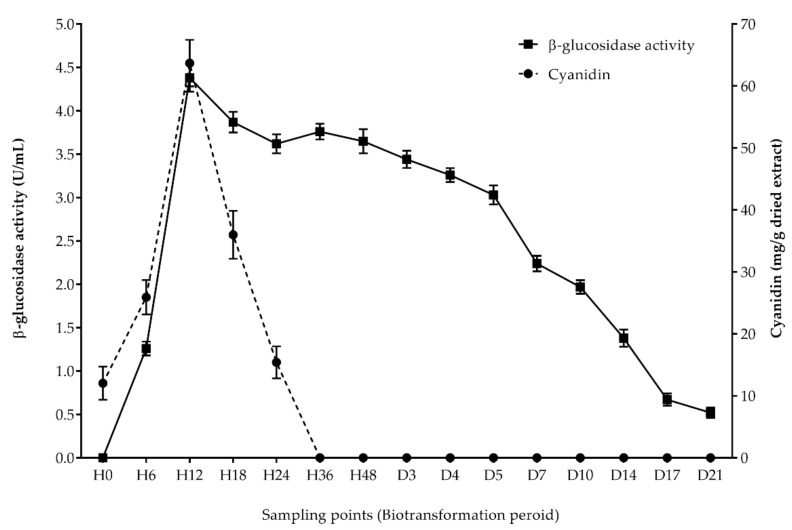
The correlation of cyanidin content with the β-glucosidase activity of selected BGPLs.

The anthocyanin extracts were prepared after 0, 6, 12, 18, 24, 36, and 48 h, as well as 3, 5, 7, 10, 14, 17, and 21 days, of the biotransformation process by the selected BGPL, *Lactobacillus* FR 332. Then, all extracts were analyzed for cyanidin-3-glucoside and cyanidin content using RP-HPLC. The results demonstrated that the level of cyanidin-3-glucoside was dramatically decreased after H0 (0 h) until H48 (48 h) and was not detectable after 3 days of the biotransformation process, as shown in Figure 1. Additionally, the amount of cyanidin was increased until H12 (12 h) and then decreased until not detectable after 36 h of the biotransformation process. Interestingly, the highest cyanidin level corresponded to the highest activity of β-glucosidase enzyme produced from *Lactobacillus* FR 332 after 12 h of the biotransformation process, as shown in Figure 2.

### 3.3. Effect of Biotransformed Anthocyanin on In Vitro Antioxidant Activities

**Figure 3:**
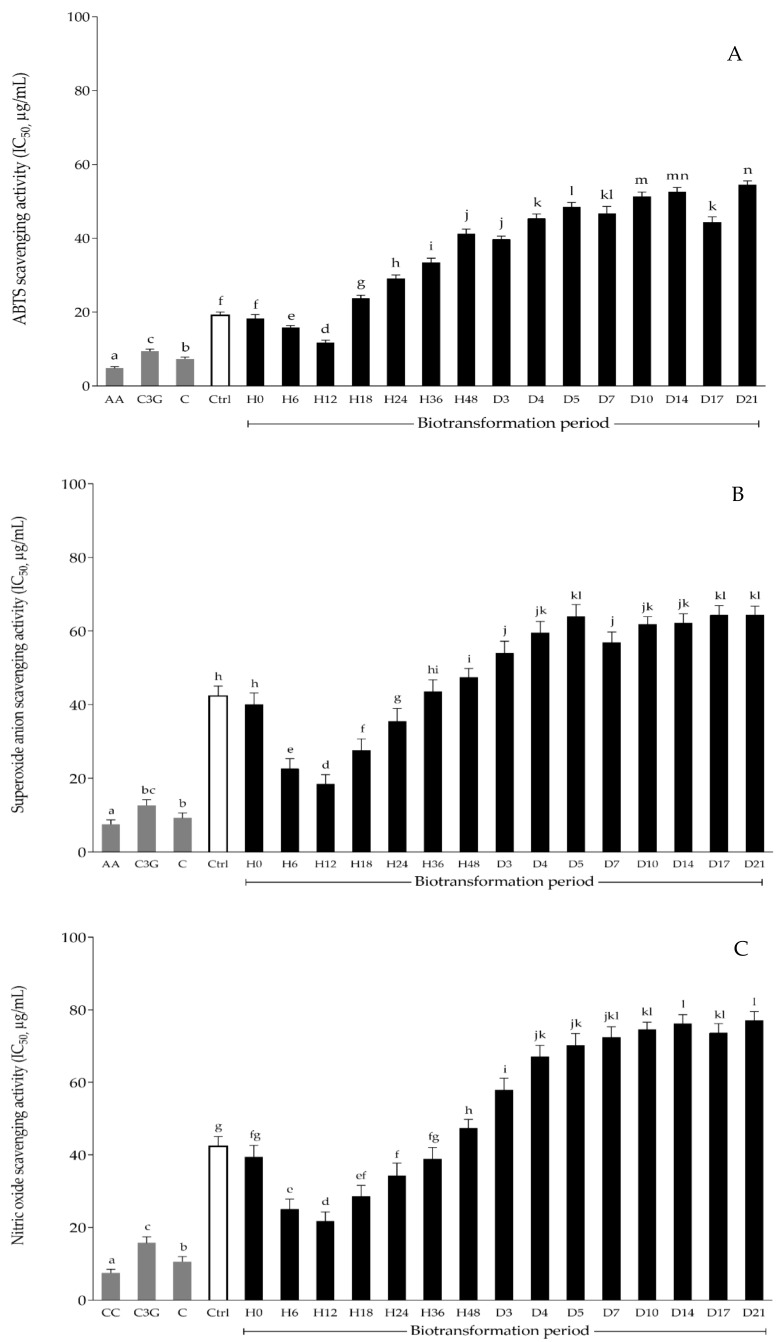
In vitro antioxidative activity of biotransformed KGLP extracts: (**A**) scavenging effect on ABTS; (**B**) scavenging effect on superoxide anions; (**C**) scavenging effect on nitric oxide. AA; l-ascorbic acid, C3G; cyanidin-3-glucoside, C; cyanidin, CC; curcumin, Ctrl; control (without BGPL). Different letters indicate significant statistical differences (*p* < 0.05).

Three in vitro antioxidant assays: ABTS scavenging, superoxide anion scavenging, and nitric oxide scavenging, were performed to investigate the free-radical scavenging capacity and antioxidant activity of the selected anthocyanin extracts, KGLP extract, and the biotransformed KGLP extract at various sampling points, as mentioned previously. The results showed that each of the collected anthocyanin extracts at different biotransformation times exhibited antioxidant activities through all assays, as shown in Figure 3A–C. Similar trends were observed in all in vitro antioxidative assays. The biotransformed anthocyanin extract collected after 12 h (H12) of the biotransformation process exhibited the significantly highest (*p* < 0.05) in vitro antioxidant activity compared to other biotransformed anthocyanin extracts in terms of the ABTS scavenging, superoxide anion scavenging, and nitric oxide scavenging, with IC_50_ values of 11.78 ± 0.67, 18.50 ± 2.54, and 21.73 ± 2.54 µg/mL, respectively. Compared to the anthocyanin extract without biotransformation (Ctrl), the biotransformed samples at 18 h, 36 h, and 48 h had significantly lower (*p* < 0.05) inhibition effects on ABTS scavenging, superoxide anion scavenging, and nitric oxide scavenging, respectively. According to the in vitro antioxidant activity results and the cyanidin-3-glucoside and cyanidin contents, anthocyanin extracts at the sampling points after 0, 12, and 24 h of the biotransformation process were selected to investigate the colorectal chemoprevention and immunization in HT-29 colorectal adenocarcinoma cells.

### 3.4. Effect of Biotransformed Anthocyanin on Colorectal Chemoprevention and Immunization Potential in HT-29 Cells

**Figure 4:**
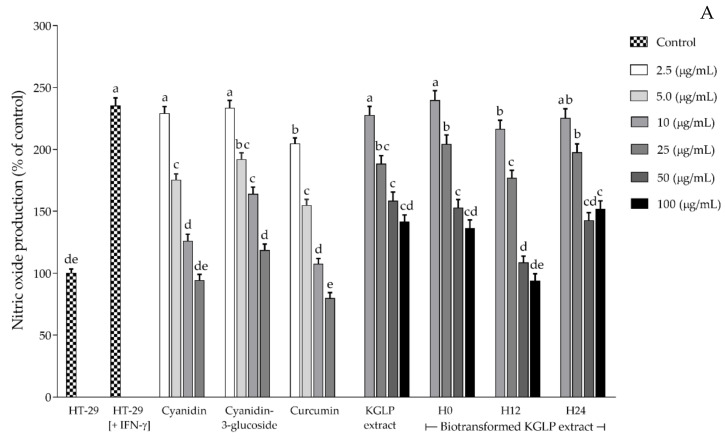

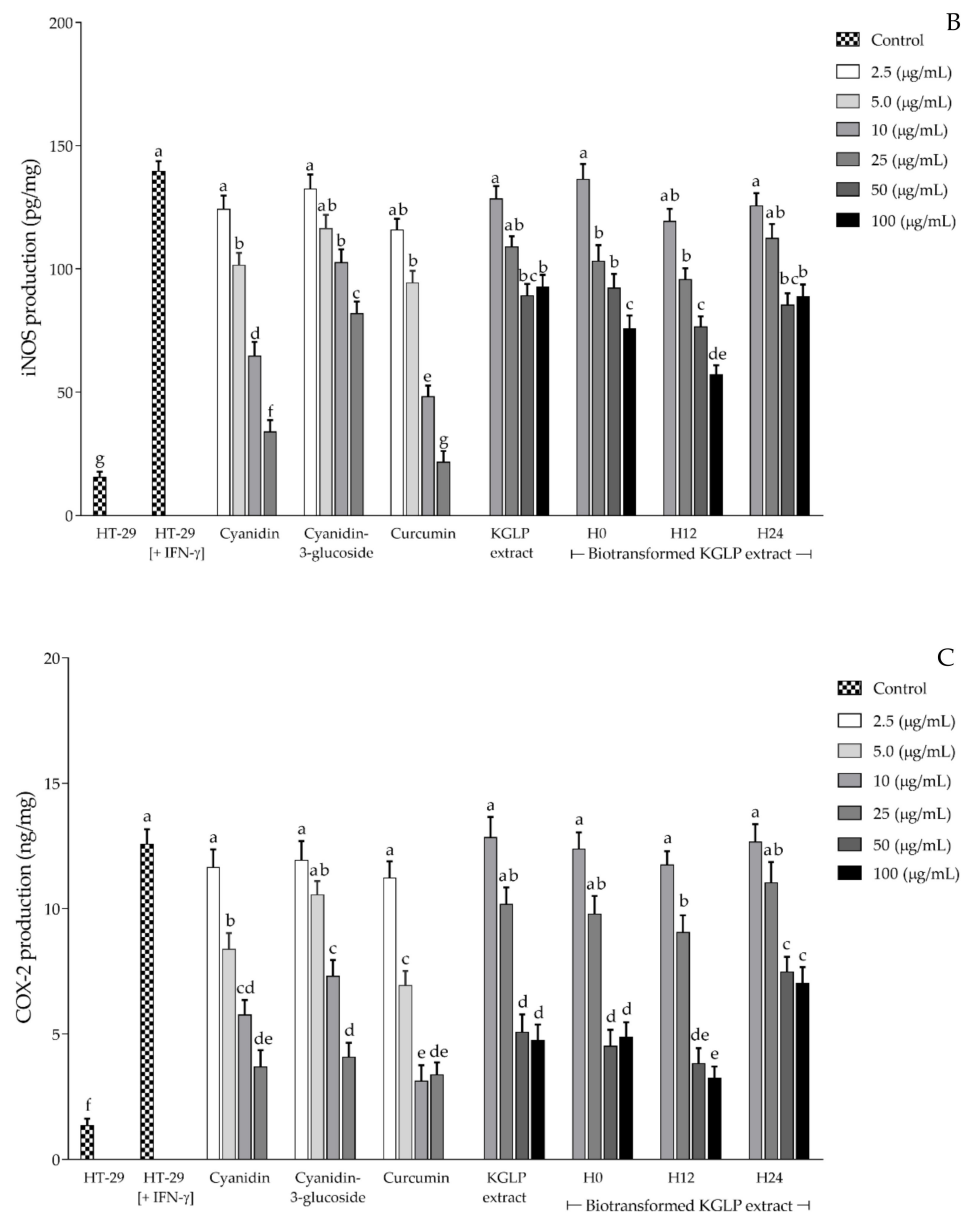
Effects of KGLP extract and biotransformed KGLP extracts on (**A**) nitric oxide production, (**B**) iNOS production, and (**C**) COX-2 production in HT-29 colorectal cells. Different letters indicate significant statistical differences (*p* < 0.05).

The three selected biotransformed anthocyanin extracts from KGLP after 0, 12, and 24 h of the biotransformation process were investigated for their anti-inflammatory and immunomodulatory activities. The effects on nitric oxide, iNOS, COX-2, IL-6, and IL-10 production were determined using HT-29 cells induced by IFN-γ. Cyanidin, cyanidin-3-glucoside, and curcumin were used as the positive controls, and KGLP extract without biotransformation was used as the control. The effect of biotransformed anthocyanin extracts on nitric oxide, iNOS, COX-2, IL-6, and IL-10 production following IFN-γ stimulation, were examined 12 h after incubation. The results showed that nitric oxide, iNOS, and COX-2 were reduced in a concentration-dependent manner without exerting cytotoxicity, as shown in Figure 4A–C. The biotransformed anthocyanin extracts H0 and H24 at 25, 50, and 100 µg/mL and H12 at 10, 25, 50, and 100 µg/mL exerted a significant (*p* < 0.05) inhibitory effect on nitric oxide and iNOS production. Additionally, H0 and H24 at 50 and 100 µg/mL and H12 at 25, 50, and 100 µg/mL exerted a significant (*p* < 0.05) inhibitory effect on COX-2 when compared to the IFN-γ-stimulated HT-29 cells. The results revealed that H12 showed the significantly highest (*p* < 0.05) inhibition activity on nitric oxide, iNOS, and COX-2 production from IFN-γ-stimulated HT-29 cells among the other compared biotransformed samples (H0 and H24) and the KGLP extract without biotransformation. However, the inhibition activity on nitric oxide, iNOS, and COX-2 of all anthocyanin extracts was lower than the standards curcumin (a potent anti-inflammatory compound), cyanidin, and cyanidin-3-glucoside at the same concentrations.

**Figure 5:**
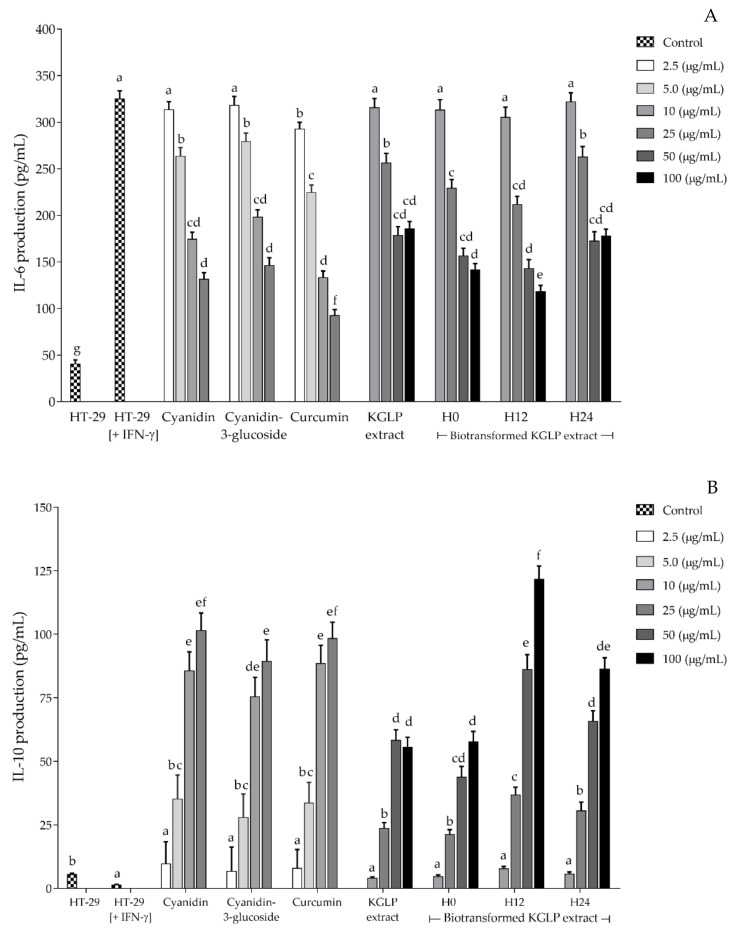
Effects of KGLP extract and biotransformed KGLP extracts on (**A**) IL-6 production and (**B**) IL-10 production in HT-29 colorectal cells. Different letters indicate significant statistical differences (*p* < 0.05).

The effects of biotransformed KGLP extract on IL-6 and IL-10 production in HT-29 colorectal cells are shown in Figure 5A,B, respectively. Both the KGLP extract and the selected biotransformed KGLP extracts exhibited colorectal immunization through inhibitory effects on proinflammatory cytokine IL-6 production and promoted anti-inflammatory cytokine IL-10 production in IFN-γ-stimulated HT-29 cells. The H12 biotransformed KGLP extract at the concentration of 100 µg/mL had significantly lower (*p* < 0.05) proinflammatory cytokine IL-6 production than the KGLP extract and two other biotransformed KGLP extracts. The H12 and H24 biotransformed KGLP extracts (25, 50, and 100 µg/mL) had significantly higher (*p* < 0.05) anti-inflammatory cytokine IL-10 production than the KGLP extract without biotransformation at the same concentration. The H12 biotransformed KGLP extract had the highest IL-10 production among the biotransformed extract samples, while IL-10 production in the H0 biotransformed KGLP extract was not significantly (*p* > 0.05) different from the KGLP extract without biotransformation.

## 4. Discussion

Anthocyanins belong to the flavonoid class of compounds; they are secondary metabolites with a flavonoid structure containing a positive charge at the C-1 position of the molecule [7], occurring widely in various colored fruits, flowers, and plants. Thai purple rice varieties (Khao’ Niaw Dam or Khao’ Gam) are widely grown across the country, especially in the highlands or mountainous areas in the north of Thailand. According to the biodiversity in the north of Thailand and the global trend of using natural active pharmaceutical ingredients (NAPIs), this research aimed to study the biotransformation of indigenous northern Thai purple rice using β-glucosidase-producing *Lactobacillus* (BGPL) to increase the bioactive compounds and their bioactivity for colorectal chemoprevention and immunization. Among the five indigenous northern Thai purple rice cultivars, KGLP contained the highest levels of cyanidin-3-glucoside and peonidin-3-glucoside, the major anthocyanins found in purple rice, followed by KGB, KGT, KGPY, and KGCR. Chiang Mai black rice cultivars were reported to contain cyanidin-3-glucoside, peonidin 3-glucoside, and cyanidin [25]. Additionally, Thai black rice Khao’ Hom Nin was reported to be composed of two major anthocyanins, cyanidin 3-glucoside and peonidin 3-glucoside [37]. As a result, KGLP was selected as the substrate for the kinetic study of the biotransformation process using the selected BGPL, *Lactobacillus* FR 322.

After 12 h of the biotransformation process, the highest activity of β-glucosidase enzyme produced from *Lactobacillus* FR 332 was observed, corresponding to the conversion of cyanidin and cyanidin-3-glucoside content during the biotransformation process. KGLP contained high cyanidin-3-glucoside or cyanidin 3-*O*-beta-d-glucoside content, i.e., with beta-d-glucose at the C-3 position in its molecular structure [7]. Our study demonstrated that β-glucosidase is produced from *Lactobacillus* FR 332, a degrading enzyme which catalyzes the hydrolysis of the glycosidic bond at the C-3 position of cyanidin-3-glucoside to free aglycone cyanidin and glucose. Cyanidin-3-glucoside can be hydrolyzed to its corresponding aglycones by enzymes in the small intestine and further degraded to phenolic compounds by the gut microbiota. The cleavage of the heterocyclic flavylium ring, which contains a positive charge at C-1, initiates the microbial catabolism of cyanidin-3-glucoside, which is followed by dehydroxylation or decarboxylation [38]. *Lactobacillus* and *Bifidobacterium* (*L. paracasei*, *B. lactis*, and *B. dentium*) have been studied for their ability to produce β-glucosidase [39,40]. The kinetic fermentation of anthocyanin from jussara fruit by β-glucosidase- and α-galactosidase-producing bacteria was investigated. The results showed that the enzymes had the highest activities after a 12 h fermentation period, with the percentage conversion of cyanidin-3-glucoside and cyanidin-3-rutinoside reaching 100% and 58% at 48 h, respectively [40]. Various probiotics have been studied for the biotransformation of mulberry anthocyanin. *L. plantarum* GIM 1.35 exhibited the highest β-glucosidase activity (1.20 ± 0.047 U/mL) after 12 h of fermentation, and the main biotransformation products were chlorogenic acid, crypto-chlorogenic acid, caffeic acid, and ferulic acid [41]. An *L. plantarum* strain was used to study the biotransformation of anthocyanin glucosides in wine solution. The highest β-glucosidase activity was observed after 72 h of fermentation, and the enzyme activity increased in the presence of anthocyanin glycosides [42]. It is possible that the type of anthocyanin, which is associated with various residual sugars, carbon sources and bacterial species, has an effect on enzyme activity. In humans and animal models, the absorption and urine excretion of intact anthocyanidin glycosides were shown to be very low, ranging from 0.016–0.11% of the dose. In addition, anthocyanin recovery in feces is extremely low. These findings suggest that anthocyanins undergo significant biotransformation upon oral consumption and absorption [43].

Biotransformed KGLP showed potent antioxidant activity in terms of scavenging ABTS, superoxide anions, and nitric oxide free radicals in our study. The scavenging effect on ABTS is generally used to test the preliminary radical-scavenging activity of compounds and plant extracts. This method is an excellent tool for determining the antioxidant activity of hydrogen-donating mechanisms and chain-breaking antioxidants [44]. The superoxide anion is a reactive oxygen species (ROS), while nitric oxide is a reactive nitrogen species (RNS). The superoxide anion and nitric oxide play various pathological roles in almost every animal species [45]. IL-6 is a proinflammatory cytokine involved in immune response, inflammation, and hemopoiesis. It is produced by normal cells (monocytes and macrophages) and tumor tissues (breast, prostate, colorectal, and ovarian cancer) [46]. IL-6 may also play a role in a variety of features of tumor behavior, including apoptosis, proliferation, migration, and invasion, as well as angiogenesis and metastasis [47]. IL-10 is an anti-inflammatory cytokine due to its inhibitory effect on T-helper cell cytokine production. It is generated by nearly all leukocytes and various human tumor cells, including those found in the breast, kidney, colon, and pancreas, as well as in malignant melanomas and neuroblastomas [46]. IL-10 may be involved in the suppression of tumor-promoting inflammatory mediators and the regulation of tumor angiogenesis [48,49].

It is proposed here that the strong inhibition effects of KGLP anthocyanin extract prepared from 12 h of biotransformation on nitric oxide, iNOS, and COX-2 production in IFN-γ-stimulated HT-29 cells is mainly due to the high content of cyanidin. Cyanidin has a lower molecular weight (287.24 g/mol) and is less hydrophilic (log P = 3.05) than cyanidin-3-glucoside (449.4 g/mol and log P = 0.39) [50]. Thus, it is possible that cyanidin can penetrate through the cell membrane due to its greater lipophilicity compared to cyanidin-3-glucoside. Cyanidin is the hydrolyzed product of cyanidin-3-glucoside, in addition to other metabolites produced by colonic microbiota such as protocatechuic acid (PCA), 2,4,6-trihydroxybenzaldehyde, 2,4,6-trihydroxybenzoic acid, vanillic acid, and ferulic acid [38,51]. Cyanidin-3-glucoside and its metabolites, including cyanidin, PCA, vanillic acid, and ferulic acid, have been reported to upregulate the antioxidant enzyme system by boosting the activities of manganese-dependent superoxide dismutase (MnSOD) and glutathione (GSH) [52]. They also downregulate the pro-oxidant system by lowering the expression of COX-2 and iNOS, thereby inhibiting the production of ROS/RNS [52,53,54]. Cyanidin-3-glucoside and cyanidin were reported to downregulate the proinflammatory mediator nitric oxide, as well as proinflammatory cytokines TNF-α, IL-1β, IL-6, and IFN-γ in 2,4,6-trinitrobenzenesulfonic acid (TNBS)-induced colitis mice and lipopolysaccharide (LPS)-stimulated Caco-2 cells [55]. Anthocyanin-rich bilberry extract was found to lower the levels of proinflammatory cytokines IFN-γ and TNF while increasing the levels of Th17 cell-specific cytokine IL-22 and immunoregulatory cytokine IL-10 in ulcerative colitis patients [56].

## 5. Conclusions

KGLP biotransformed by β-glucosidase-producing *L. plantarum* FR332 produced the highest cyanidin content via deglucosidation of cyanidin-3-glucoside after 12 h of biotransformation. The anthocyanin extract after 12 h of biotransformation exhibited the most potent in vitro antioxidative and anti-inflammatory activity by inhibiting cyclooxygenase-2 (COX-2), nitric oxide, and inducible nitric oxide synthase production in IFN-γ-stimulated colon adenocarcinoma (HT-29) cells without exerting cytotoxicity. Additionally, the anthocyanin extract after 12 h of biotransformation also had a potent inhibitory effect on proinflammatory IL-6 secretion and an inducive effect on anti-inflammatory cytokine IL-10 secretion. The current results highlight the health benefits of the anthocyanin extract from KGLP biotransformed for 12 h by *L. plantarum* FR332 as a natural active pharmaceutical ingredient (NAPI) for colorectal chemoprevention and immunization.

## Figures and Tables

**Table 1:** Anthocyanin contents of five selected northern Thai purple rice cultivars.

Purple Rice Cultivars	Amount of Anthocyanin (mg/100 g Dried Weight)			
	Cyanidin-3-glucoside	Peonidine-3-glucoside	Cyanidin	Peonidin
Khao’ Gam Chiang Rai (KGCR)	37.29 ± 2.43 ^d^	16.76 ± 2.07 ^d^	ND	ND
Khao’ Gam Pha Yao (KGPY)	35.62 ± 2.28 ^d^	23.49 ± 1.85 ^c^	ND	ND
Khao’ Gam Leum-Phua (KGLP)	192.19 ± 3.10 ^a^	84.54 ± 2.78 ^a^	7.67 ± 0.62 ^a^	ND
Khao’ Gam Thor (KGT)	67.63 ± 2.65 ^c^	26.35 ± 2.15 ^c^	3.54 ± 0.48 ^c^	ND
Khao’ Gam Boung (KGB)	108.49 ± 2.78 ^b^	57.83 ± 2.19 ^b^	5.31 ± 0.45 ^b^	ND

All values are expressed as means ± standard deviation (SD; *n* = 3). Different letters in each column indicate a significant difference (*p* < 0.05). ND = not detectable.

## Data Availability

The original contributions generated for this study are included in the article; the data presented in this study are available on request from the corresponding author.

## Supplementary Materials

The following supporting information can be downloaded at: https://www.mdpi.com/article/10.3390/antiox11020305/s1, Figure S1: Cell viability assessed by the cell proliferation reagent WST-1 in 72 h ofHT-29 cultured without IFN-γ, Figure S2: Cell viability assessed by the cell proliferation reagentWST-1 in 72 h of HT-29 cultured with IFN-γ.
