# Supplementary material for: Enhancement of the Colorectal Chemopreventive and Immunization Potential of Northern Thai Purple Rice Anthocyanin Using the Biotransformation by β-Glucosidase-Producing Lactobacillus
Source: Antioxidants (Basel). 2022 Feb 2;11(2):305. doi: 10.3390/antiox11020305 (PMC8868395; doi:10.3390/antiox11020305)
Supplement: Supplementary file 1 [file antioxidants-11-00305-s001.zip › antioxidants-1554782-supplementary.pdf]

antioxidants

MDPI

Article

Enhancement of the Colorectal Chemopreventive and Immunization Potential of Northern Thai Purple Rice Anthocyanin Using the Biotransformation by β-Glucosidase-Producing *Lactobacillus*

Sasithorn Sirilun [1,2], Chaiyavat Chaiyasut [1,2], Thanawat Pattananandecha [1,3,4], Sutasinee Apichai [1,3,4], Jakkapan Sirithunyalug [1,3], Busaban Sirithunyalug [1,3,*] and Chalermpong Saenjum [1,3,4,*]

[1] Department of Pharmaceutical Sciences, Faculty of Pharmacy, Chiang Mai University, Chiang Mai 50200, Thailand; ssirilun@gmail.com (S.S.); chaiyavat@gmail.com (C.C.); thanawat.pdecha@gmail.com (T.P.); sutasinee.apichai@gmail.com (S.A.); jakkapan.s@cmu.ac.th (J.S.)

[2] Innovation Center for Holistic Health, Nutraceuticals and Cosmeceuticals, Faculty of Pharmacy, Chiang Mai University, Chiang Mai 50200, Thailand

[3] Center of Excellence for Innovation in Analytical Science and Technology for Biodiversity-Based Economic and Society (I-ANALY-S-T_B.BES-CMU), Chiang Mai University, Chiang Mai 50200, Thailand

[4] Multidisciplinary Approaches to Lanna Fermented Foods and Biological Resources Research Unit, Sciences and Technology Research Institute (STSI), Chiang Mai University, Chiang Mai 50200, Thailand

[*] Correspondence: busaban.s@cmu.ac.th (B.S.); chalermpong.s@cmu.ac.th (C.S.); Tel.: 66-81-990-7971 (B.S.); +66-89-950-4227 (C.S.)

Supplementary Materials

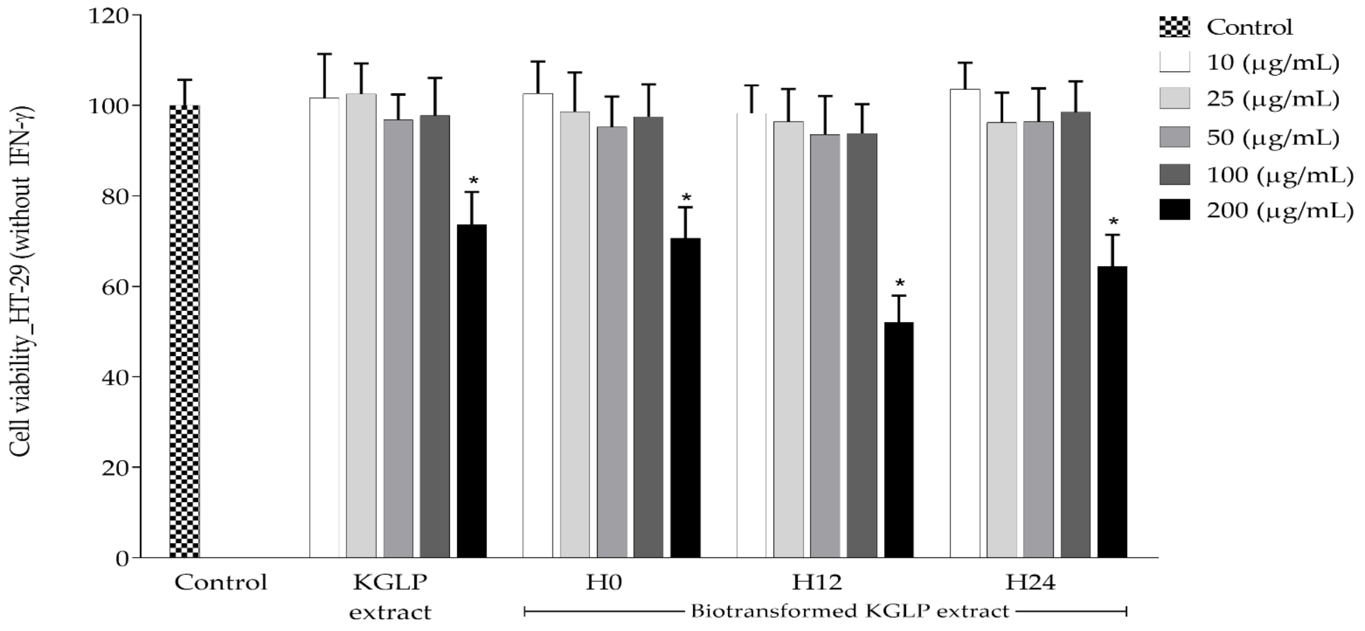

Figure S1. Cell viability assessed by the cell proliferation reagent WST-1 in 72 h of HT-29 cultured without IFN-γ. * = $p < 0.05$.

SENSITIVE

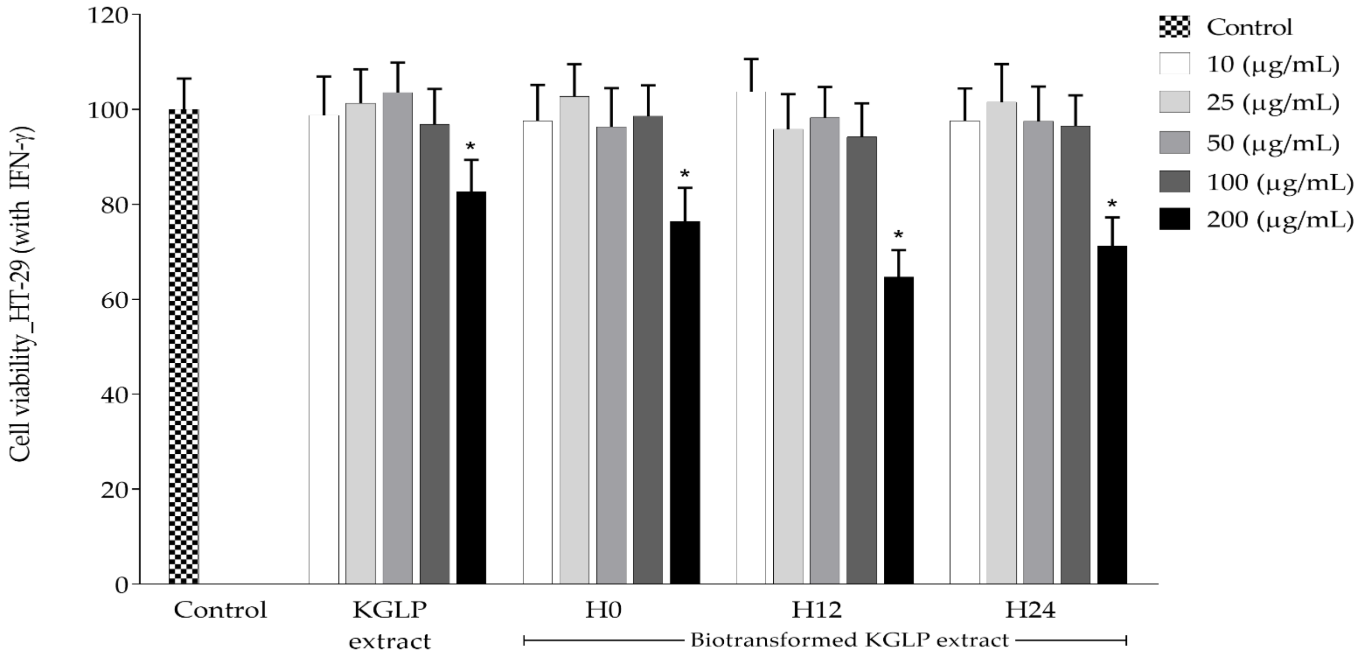

Figure S2. Cell viability assessed by the cell proliferation reagent WST-1 in 72 h of HT-29 cultured with IFN-γ. * = $\rho < 0.05$.

SENSITIVE
